# Schistosomiasis and Soil Transmitted Helminths Distribution in Benin: A Baseline Prevalence Survey in 30 Districts

**DOI:** 10.1371/journal.pone.0162798

**Published:** 2016-09-19

**Authors:** Pelagie M. Boko, Moudachirou Ibikounle, Ablawa Onzo-Aboki, Jean-Jacques Tougoue, Yollande Sissinto, Wilfrid Batcho, Dorothe Kinde-Gazard, Achille Kabore

**Affiliations:** 1 National Control Program of Communicable Diseases, Ministry of Health of Benin, 01BP882 Cotonou, Benin; 2 Faculty of Sciences and Technique, University of Abomey-Calavi, 01BP526 Cotonou, Benin; 3 Research Triangle Institute, Washington, District of Columbia, United States of America, 3040 Cornwallis Road, Research Triangle Park, NC 27709, United States of America; 4 Faculty of Health Sciences, University of Abomey-Calavi, 01BP 526 Cotonou, Benin; Universidade de Aveiro, PORTUGAL

## Abstract

In 2013, Benin developed strategies to control neglected tropical diseases and one of the first step was the disease mapping of the entire country in order to identify endemic districts of schistosomiasis and soil transmitted helminths (STH). This study was carried out in 30 of the 77 districts of Benin. Of these 30 districts 22 were previously treated for Lymphatic Filariasis (LF) using the Ivermectin and Albendazole combination. In each district, five schools were selected and 50 children aged 8 to 14 years were sampled in each school, making a total of 250 children sampled in the district. The schools were selected mainly according to their proximity to lakes or any bodies of water that were likely to have been used by the children. Samples of faeces and urine were collected from each pupil. Urinary schistosomiasis was identified using the urine filtration technique while STH and intestinal schistosomiasis were identified through the Kato Katz method. Overall a total of 7500 pupils were surveyed across 150 schools with a gender ratio of 1:1. Hookworm was identified in all 30 districts with a prevalence ranging from 1.2% (95%CI: 0.0–2.5) to 60% (95%CI: 53.9–66.1). *Ascaris lumbricoides* was detected in 19 districts with a prevalence rate between 1% (95%CI: 0.0–2.2) and 39% (95%CI: 32.9–45.0). In addition to these common STH, *Trichuris trichiura*, *Enterobius vermicularis* and *Strongyloides stercoralis* were found at low prevalence. Only 16 districts were endemic to *Schistosoma mansoni*, while 29 districts were endemic to *S*. *haematobium*. The *S*. *haematobium* prevalence ranged from 0.8% (95% CI: 0.0–1.9) to 56% (95% CI: 50.2–62.5) while the prevalence of *S*. *mansoni* varied from 0.4% (95%CI: 0.0–1.2) to 46% (95% CI: 39.8–52.2). The 22 districts, where LF was successfully eliminated, still require mass drug administration (MDA) of albendazole indicating that school-based MDA would be needed even after LF elimination in districts co-endemic to LF and STH in Benin.

## Introduction

Schistosomiasis and soil-transmitted helminthiasis (STH) are the most widespread parasitic neglected tropical diseases in the world. These infections are poverty-related and particularly abundant among people with limited access to safe water, sanitary facilities or adequate health facilities [1–3].

Schistosomiasis, is water-associated vector-borne disease caused by parasitic blood flukes, known as schistosomes with at least 200 million people affected worldwide [4–6]. According to previous estimates, the disease causes the annual loss of between 1.7 and 4.5 million disability adjusted life years (DALYs) [1]. Of the 78 endemic countries, 39 are located in Africa and it is estimated that around 85 to 90% of the people at risk live in Sub-Saharan Africa [5,7] making schistosomiasis the most prevalent neglected tropical disease in Sub-Saharan Africa. In comparison to malaria, schistosomiasis is the second most important parasitic disease in the majority of the African nations when comparing the number of infected people [8]. The morbidity due to schistosome infection is dependent on whether it is intestinal or urinary schistosomiasis. *S mansoni* infection has been associated with diarrhoea, blood in stool and hepatosplenomegaly while S haematobium infection is associated with haematuria, dysnuria and major hydronephrosis [9] as well as bladder cancer although data are scarce in support of this. Schistosomiasis complications can lead to death [10].

As with schistosomiasis, most of the people affected with STH are in Sub-Saharan Africa [3,11–13]. STH are commonly known as intestinal worms and three main parasites are frequently associated with STH including *Ascaris lumbricoides*, whipworms (*Trichuris trichiura*) and Hookworms (*Ancylostoma duodenale* and *Necator americanus*). Most of the STH are from faecal-oral contamination or by trans-cutaneous penetration through unprotected skin, suggesting a lack of personal hygiene and poor sanitation in affected populations. STH infection can lead to anaemia caused by the parasites, intestinal obstruction and reduced vitamin A absorption, resulting in the stunted growth of children affected [14,15]. Impairment of cognitive development in young children associated to poor educational outcomes is frequently reported as is morbidity due to STH [16–19].

An escalation in schistosomiasis and STH control has been observed in the last decade. The control of these neglected tropical diseases (NTD) has become a priority for many governments and has the support of donors as international organizations following the World Health Assembly resolution in 2001 [20]. In Benin, the control of preventive chemotherapy NTD is progressively being scaled up following WHO guidelines. Instead of sporadic treatment with a Praziquantel and Albendzole combination that would normally be provided to school age children by the National Public Health department to reduce the burden of schistosomiasis and STH, a National control Program has been implementing mass drug administration in districts with confirmed cases. However the misconception that schistosomiasis is not a disease [21] make the population reluctant to seek treatment even in the presence of explicit symptoms such as haematuria. For most people in rural areas, schistosomiasis is considered to be a sign of puberty for boys rather than a disease. STH control is also facing challenges in communities in which the use of toilets is forbidden by local traditions. Nevertheless, in order to treat people in each district based on prevalence and achieve control of these NTDs by 2020, the national control program of communicable diseases with the support of RTI ENVISION intends as a starting point to assess the distribution of STH and schistosomiasis in all districts of Benin (West Africa). This study aimed to identify the baseline prevalence of both schistosomiasis and STH in 30 districts of Benin prior to the implementation of control strategies.

## Materials and Methods

### Ethical considerations

Ethical approval was granted from the National Ethic Committee for Health Research, (Comite National d’Ethique pour la Recherche en Sante; Ministere de la Sante CNERS-MS) under the authorisation reference 009/CNERS-MS. With the approval of the CNERS-MS, written consent was obtained from each school’s head teacher and sometimes from the chief of each village on behalf of school parents. In cases where those that were to give authorisation were unable to read and write, a detailed verbal explanation of the form was given so that informed consent was guaranteed. Two copies of the written consent form were therefore signed and dated. The person giving consent kept one copy with the second copy being returned to the National Control Program of Communicable Diseases. In some districts where parent and teacher associations exist, the head of the association and school’s head teacher would be responsible for providing formal approval using the consent form. No sample was collected until the consent form was signed.

Participants found with a high intensity of schistosomiasis infection were directed to a health centre in order to receive appropriate treatment before the mass drug administration scheduled for the following year.

### Study sites and sample collection

**Fig 1:**
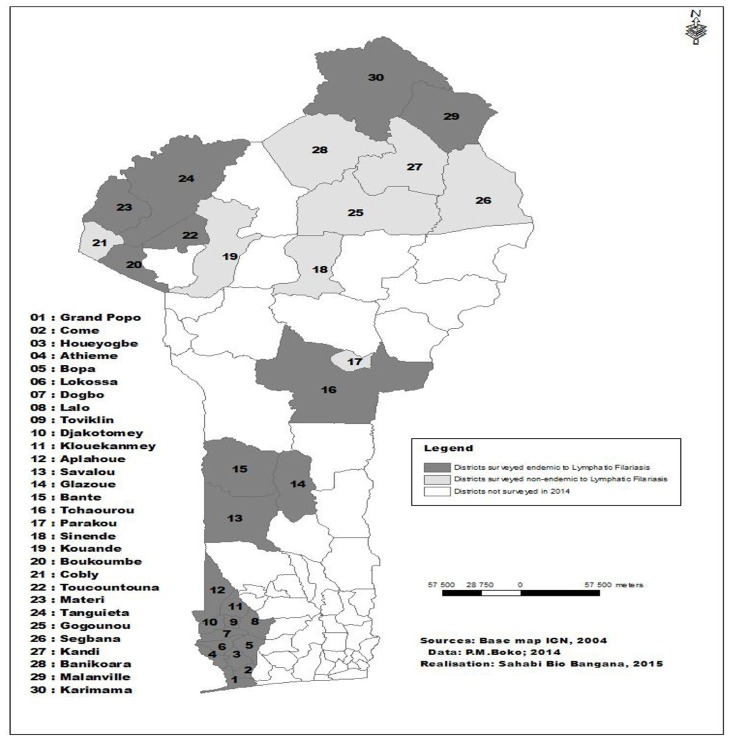
Districts surveyed during Schistosomiasis and soil transmitted helminths mapping in 2014. The surveyed districts endemic to lymphatic filariasis, have received six rounds of LF-MDA in community. Elimination of LF was confirmed following transmission assessment survey and MDA was stopped in these districts in 2013.

The study was carried out in early 2014 in 30 districts selected in five departments of Benin (Fig 1). The country is divided into 12 departments (political subdivisions), which are further divided into 77 districts. These districts are further divided into 545 sub-districts with each sub-district having at least one public school. The geographical location of each school surveyed including the departments’ Universal Transverse Mercator coordinates is provided in S1 Table. The districts surveyed in this study were located in differing hydro-geographic systems with a rainfall spread that increases from the south to the north. In the northern departments (Atacora, Borgou and Alibori), the annual rainfall varies between 900 mm and 1200 mm with numerous lakes and rivers feeding the region. In the southern departments (Collines, Mono and Couffo), the annual rainfall varies from 800 mm to 1200 mm. Of these 30 districts 22 were previously treated for Lymphatic Filariasis (LF) using the Ivermectin and Albendazole combination. In each district, five schools were selected based on their proximity to a river or bodies of water where snails, suspected to be the vector of schistosomiasis in the area, are present and where children are often seen swimming. In each school, 25 girls and 25 boys aged between 8 and 14 years were randomly selected. Eligible boys and girls who agreed to participate in the study were divided into two lines by gender. The total number in each line was divided by 25 to determine the sampling interval. This number was rounded up to the closest integer. Then counting each child from the start of the line every child with a position which was a multiple of the sampling interval was selected for the study. We selected this age group not only for convenience but also because children are generally more active at this age, more likely to go swimming and also more likely to be exposed to STH [22]. The children were given two containers each in order to provide urine and faeces samples. The samples were collected within an hour. The target sample size was estimated with a desired precision of 5%, confidence level of 95% and an expected prevalence of 20% based on the country report following a questionnaire-based survey in 2003.

### Detection of schistosomiasis and STH

The urine samples were tested for the presence of blood using reagent strips Uricolor^®^. In addition 10ml of each urine sample were filtered through a Nytrile filter (12–14 μm pore size) and stained with 10% Lugol [23] in order to identify urinary schistosomiasis infection. The eggs were double-counted within an hour following the filtration by two lab-technicians. The intensity of the infestation was estimated as number of eggs of *S*. *haematobium* per 10 ml of urine and classified as either light or high infection[24].

The faeces samples were analysed using the Kato Katz technic in which 41.7 mg of faeces was filtered through a nylon mesh and covered with cellophane previously soaked in 50% Malachite Green [25]. The slides were observed under a microscope by two technicians and their results were validated by a supervisor. Hookworm eggs were counted within 30 min after slides were prepared before they cleared. All the other STH eggs and the *S*. *mansoni* eggs were counted 24 hours later. The intensity of the infestation was estimated in terms of the number of eggs per gram (EPG) of faeces for the designated parasite and classified as either light, moderate or intense.

The mapping of the entire country had been carried out over a three years period. The current study was limited to the 30 districts out of the 77 of the country due to resources constraints though the following year the remaining districts were to be surveyed.

Prior to sample collection, all lab-technicians and their supervisors received refresher training in the Kato Katz, urine filtration technique and the standard operating procedure to be followed in order to ensure normalisation of data collected. Each team was given a training module which included images of the various parasites expected.

For quality control purpose, 10% of the collected slides in each district were examined by an independent team of biologists and parasitologists.

### Statistical analysis

R Core Team (R Version 3.2.2–2015) software was used for data processing. The multiple comparison test chi2 proportions was used to compare the prevalence by departments. The chi-square test comparison of two proportions was also used to compare the prevalence of STH and schistosomiasis within each district. The Fisher exact method of maximum likelihood and calculation of confidence intervals was used to calculate odds ratios by gender in each of the districts. Average of prevalence histograms were obtained after a least significant difference test on an analysis of variance with department as a factor. The bands of the histogram affected by the same letter are averages not significantly different prevalence and those sharing different letters represent averages significantly different prevalence. The Epitools R package for Epidemiologic Data and Graphs [26] was used to estimate the confidence intervals.

## Results

Overall 7500 pupils in regular attendance at school were surveyed in 150 schools of the 30 districts (Table 1). The gender ratio was 1:1. At least one type of STH was identified in all 30 districts surveyed and one type of schistosomiasis was found in 29 districts. Hookworm was the most widespread STH while *S*. *haematobium* was the common form of schistosomiasis in 29 of the districts (Table 1). The p-value and the chi-square value obtained from the comparison of *S*. *haematobium* prevalence and Hookworm prevalence within a district are summarised in Table 2.

**Table 1:** Prevalence of the most common soil transmitted helminths and schistosomiasis found in 30 districts in Benin. In each column, the letter “a”, “b” or “c” indicates that the prevalence sharing similar letter are not significantly different while those sharing different letters are significantly different [p<0.0001]. **N: Total number sampled, 95%CI: 95% confidence interval for prevalence.**

				Soil Transmitted Helminths Prevalence (%)		Schistosomiasis Prevalence (%)		Lavatory Type of facilities		
Department	District	N School Surveyed	N Pupils 8–14 years	Hookworm [95%CI]	*Ascaris lumbricoides* [95%CI]	*S*. *haematobium* [95%CI]	S. *mansoni* [95%CI]	N school with lavatory	Modern	Traditional
**Alibori**	Banikoara	5	250	7.6 ^a^ [4.3–10.9]	0.0 [0.0–0.0]	25.2 ^a^ [19.8–30.6]	0.0 ^a^ [0.0–0.0]	5	4	1
	Gogounou	5	250	10.4 ^a^ [6.6–14.2]	0.0 [0.0–0.0]	6.4 ^b^ [3.4–9.4]	0.0 ^a^[0.0–0.0]	5	5	0
	Malanville	5	250	2.8 ^b^ [0.7–4.8]	15.0 [10.6–19.4]	32.0 ^a^ [26.2–37.8]	0.4 ^a^ [0.0–1.2]	5	5	0
	Karimama	5	250	1.2 ^b^[0.0–2.5]	6.0[3.1–8.9]	9.2 ^b^[5.6–12.8]	0.0 ^a^ [0.0–0.0]	5	5	0
	Segbana	5	250	14.0 ^a^ [9.7–18.3]	3.0 [0.9–5.1]	2.0 ^b^ [0.3–3.7]	2.0 ^a^[0.3–3.7]	5	5	0
	Kandi	5	250	4.8 ^a^ [2.1–7.4]	9.0 [5.5–12.5]	23.2 ^a^ [17.9–28.4]	0.0[0.0–0.0]	5	5	0
**Atacora**	Cobly	5	250	30.4 ^b^ [24.7–36.1]	1.0[0.0–2.2]	5.6 ^b^[2.7–8.4]	2.0 ^b^[0.3–3.7]	5	5	0
	Boukoumbe	5	250	13.2 ^a^ [9.0–17.4]	2.0 [0.3–3.7]	20.0 ^a^ [15.0–24.9]	18.4 ^a^ [13.6–23.2]	4	4	0
	Materi	5	250	10.8 ^a^ [6.9–14.6]	0.0 [0.0–0.0]	2.8 ^b^ [0.7–4.8]	6.4 ^c^ [3.4–9.4]	4	4	0
	Kouande	5	250	21.6 ^b^ [16.5–26.7]	0.0 [0.0–0.0]	11.2 ^a^ [7.3–15.1]	0.4 ^a^ [0.0–1.2]	5	5	0
	Toucountouna	5	250	4.4 ^c^ [1.9–6.9]	0.0 [0.0–0.0]	0.8 ^c^ [0.0–1.9]	0.0 ^e^ [0.0–0.0]	4	4	0
	Tanguieta	5	250	10.4 ^a^ [6.6–14.2]	2.0 [0.3–3.7]	0.8 ^c^ [0.0–1.9]	46.0 ^d^ [39.8–52.2]	5	5	0
**Borgou**	Parakou	5	250	21.2 ^a^ [16.1–26.3]	0.0 [0.0–0.0]	22.4 ^b^ [17.2–27.6]	0.4 ^a^ [0.0–1.2]	5	5	0
	Sinende	5	250	5.6 ^b^ [2.7–8.4]	8.0 [4.6–11.4]	36.4 ^a^ [30.4–42.4]	4.0 ^a^ [1.6–6.4]	5	5	0
	Tchaourou	5	250	19.2 ^a^ [14.3–24.1]	0.0 [0.0–0.0]	56.4 ^c^ [50.2–62.5]	0.8 ^a^ [0.0–1.9]	5	5	0
**Collines**	Glazoue	5	250	24.4 ^a^ [19.1–29.7]	0.0 [0.0–0.0]	6.4 ^b^ [3.4–9.4]	0.0 [0.0–0.0]	5	5	0
	Savalou	5	250	16.8 ^a^ [12.2–21.4]	0.0 [0.0–0.0]	20.0 ^a^ [15.0–24.9]	0.0 [0.0–0.0]	5	5	0
	Bante	5	250	24.4 ^a^ [19.1–29.7]	0.0 [0.0–0.0]	18.4 ^a^ [13.6–23.2]	0.0 [0.0–0.0]	5	4	0
**Couffo**	Klouekanmey	5	250	38.4^a^ [32.4–44.4]	6.0 [3.1–8.9]	24.8 ^a^ [19.4–30.1]	0.4 ^ab^ [0.0–1.2]	5	5	0
	Dogbo	5	250	1.2^c^ [0.0–2.5]	8.0 [4.6–11.4]	3.2 ^b^ [1.1–5.4]	0.0 ^a^ [0.0–0.0]	5	5	0
	Aplahoue	5	250	32.4^a^ [26.6–38.2]	0.0 [0.0–0.0]	18.8 ^a^ [13.9–23.6]	0.4 ^ab^ [0.0–1.2]	4	4	0
	Djakotomey	5	250	60.0^b^ [53.9–66.1]	21.0 [15.9–26.0]	8.4 ^b^ [4.9–11.8]	4.0 ^b^ [1.6–6.4]	4	4	0
	Lalo	5	250	29.2^a^ [23.6–34.8]	17.0 [12.3–21.7]	19.2 ^a^ [14.3–24.1]	0.0 ^a^ [0.0–0.0]	3	2	1
	Toviklin	5	250	18.4^d^ [13.6–23.2]	21.0 [15.9–26.0]	0.4 ^c^ [0.0–1.2]	0.0 ^a^ [0.0–0.0]	5	5	0
**Mono**	Bopa	5	250	8.8^a^ [5.3–12.3]	7.0 [3.8–10.2]	0.8 ^b^ [0.0–1.9]	1.6 ^a^ [0.1–3.2]	5	5	0
	Athieme	5	250	12.0 ^a^ [7.9–16.0]	4.0 [1.6–6.4]	5.6 ^a^ [2.7–8.4]	0.0 ^a^ [0.0–0.0]	5	5	0
	Come	5	250	4.0 ^b^ [1.6–6.4]	39.0 [32.9–45.0]	1.2 ^b^ [0.0–2.5]	1.2 ^a^ [0.0–2.5]	5	5	0
	Lokossa	5	250	30.4 ^c^ [24.7–36.1]	17.0 [12.3–21.7]	8.0 ^a^ [4.6–11.4]	14.8 ^b^ [10.4–19.2]	5	5	0
	Grand Popo	5	250	8.0 ^a^ [4.6–11.4]	1.0 [0.0–2.2]	0.0 ^b^ [0.0–0.0]	0.0 ^a^ [0.0–0.0]	4	4	0
	Houeyogbe	5	250	15.2 ^a^ [10.7–19.6]	12.0 [7.9–16.0]	12.0 ^a^ [7.9–16.0]	0.0 ^a^ [0.0–0.0]	5	5	0

**Table 2:** Comparison of the prevalence of the most common STH (Hookworm) to *S*.*haematobium*. **X2: Chi-square value of comparison of Hookworm and S. haematobium prevalence within each district. p-value: p value of comparison of Hookworm and S. haematobium prevalence within each district.** In each column, the letter “a”, “b” or “c” indicates that the prevalence sharing similar letter are not significantly different while those sharing different letters are significantly different.

Department	District	Hookworm Prevalence (%)	*S*. *haematobium* Prevalence (%)	p-value	X2
**Alibori**	Banikoara	7.6 ^a^	25.2 ^a^	< 0.0001	26.9
	Gogounou	10.4 ^a^	6.4 ^b^	0.107	2.1
	Malanville	2.8 ^b^	32.0 ^a^	< 0.0001	72.1
	Karimama	1.2 ^b^	9.2 ^b^	0.00013	14.6
	Segbana	14.0 ^a^	2.0 ^b^	< 0.0001	22.8
	Kandi	4.8 ^a^	23.2 ^a^	< 0.0001	33.6
**Atacora**	Cobly	30.4 ^b^	5.6 ^b^	< 0.0001	50.4
	Boukoumbe	13.2 ^a^	20.0 ^a^	0.041	3.7
	Materi	10.8 ^a^	2.8 ^b^	0.00074	11.4
	Kouande	21.6 ^b^	11.2 ^a^	0.00169	9.1
	Toucountouna	4.4 ^c^	0.8 ^c^	0.0246	5.0
	Tanguieta	10.4 ^a^	0.8 ^c^	< 0.0001	20.0
**Borgou**	Parakou	21.2 ^a^	22.4 ^b^	0.8285	0.05
	Sinende	5.6 ^b^	36.4 ^a^	< 0.0001	69.6
	Tchaourou	19.2 ^a^	56.4 ^c^	< 0.0001	72.0
**Collines**	Glazoue	24.4 ^a^	6.4 ^b^	< 0.0001	29.7
	Savalou	16.8 ^a^	20.0 ^a^	0.3558	0.6
	Bante	24.4 ^a^	18.4 ^a^	0.102	2.3
**Couffo**	Klouekanmey	38.4^a^	24.8 ^a^	0.00107	10.1
	Dogbo	1.2^c^	3.2 ^b^	0.223	1.5
	Aplahoue	32.4^a^	18.8 ^a^	0.0004	11.4
	Djakotomey	60.0^b^	8.4 ^b^	< 0.0001	145.6
	Lalo	29.2^a^	19.2 ^a^	0.00904	6.3
	Toviklin	18.4^d^	0.4 ^c^	< 0.0001	45.5
**Mono**	Bopa	8.8^a^	0.8 ^b^	0.00007	15.8
	Athieme	12.0 ^a^	5.6 ^a^	0.011	5.6
	Come	4.0 ^b^	1.2 ^b^	0.091	2.8
	Lokossa	30.4 ^c^	8.0 ^a^	< 0.0001	38.9
	Grand Popo	8.0 ^a^	0.0 ^b^	0.00001	18.8
	Houeyogbe	15.2 ^a^	12.0 ^a^	0.297	0.8

### STH distribution and intensity

The prevalence rate of Hookworm varies from 1.2% (95%CI: 0.0–2.5) to 60% (95%CI: 53.9–66.1). The highest prevalence was observed in the district of Djakotomey within the department of Couffo whereas the lowest prevalence was observed in the district of Dogbo in the southern region of Benin and in Karimama in the department of Alibori in the northern region (Table 1). In general, the intensity of the infestation was light as 83% to 100% of the children sampled had a light infestation, 1 to 1999 eggs per gram (EPG) of faeces. However, a few highly infected Hookworm cases were recorded (Table 3). The percentage of those identified with high loads of Hookworm eggs in positive cases were found in only four districts, Come (10%), Lalo (4.1%), Lokossa (1.3%) and Djakotomey (1.3%). In the district of Lokossa, up to 8441 EPG of Hookworm were found.

**Table 3:** Intensity of Hookworm and *A*. *lumbricoides* infections in districts surveyed. N: Number of positive sampled out the total (250) examined in each commune. For Hookworm intensity, Light: 1-1999EPG; Moderate: 2000-3999EPG; High: ≥4000EPG. For *A*. *lumbricoides* intensity, Light: 1-4999EPG; Moderate: 5000-49999EPG; High: ≥50000EPG.

District	Hookworm						*Ascaris lumbricoides*					
	Light		Moderate		High		Light		Moderate		High	
	N	%	N	%	N	%	N	%	N	%	N	%
**Banikoara**	19	100	0	0	0	0	25	100	0	0	0	0
**Gogounou**	26	100	0	0	0	0	14	100	0	0	0	0
**Malanville**	7	100	0	0	0	0	15	100	0	0	0	0
**Karimama**	3	100	0	0	0	0	6	100	0	0	0	0
**Segbana**	35	100	0	0	0	0	33	100	0	0	0	0
**Kandi**	12	100	0	0	0	0	9	100	0	0	0	0
**Cobly**	72	94.7	4	5.3	0	0	13	100	0	0	0	0
**Boukoumbe**	31	93.9	2	6.1	0	0	32	100	0	0	0	0
**Materi**	27	100	0	0	0	0	30	93.7	2	6.2	0	0
**Kouande**	52	96.3	2	3.7	0	0	15	93.7	1	6.2	0	0
**Toucountouna**	11	100	0	0	0	0	15	100	0	0	0	0
**Tanguieta**	26	100	0	0	0	0	12	100	0	0	0	0
**Tchaourou**	48	100	0	0	0	0	14	100	0	0	0	0
**Parakou**	52	98.1	1	1.9	0	0	17	100	0	0	0	0
**Sinende**	14	100	0	0	0	0	8	100	0	0	0	0
**Bante**	60	98.4	1	1.6	0	0	56	100	0	0	0	0
**Glazoue**	61	100	0	0	0	0	58	100	0	0	0	0
**Savalou**	36	85.7	6	14.3	0	0	11	100	0	0	0	0
**Aplahoue**	81	100	0	0	0	0	41	100	0	0	0	0
**Djakotomey**	142	94.7	6	4.0	2	1.3	17	80.9	3	14.3	1	4.7
**Dogbo**	3	100	0	0	0	0	8	100	0	0	0	0
**Klouekanmey**	94	97.9	2	2.1	0	0	4	66.7	2	33.3	0	0
**Lalo**	58	79.5	12	16.4	3	4.1	7	41.2	6	35.3	4	23.5
**Toviklin**	45	97.8	1	2.2	0	0	17	80.9	3	14.3	1	4.7
**Athieme**	25	83.3	5	16.7	0	0	3	75.0	1	25.0	0	0
**Bopa**	22	100	0	0	0	0	7	100	0	0	0	0
**Come**	9	90.0	0	0	1	10.0	27	69.2	12	30.8	0	0
**Grand Popo**	18	90.0	2	10.0	0	0	1	100	0	0	0	0
**Houeyogbe**	36	94.7	2	5.3	0	0	13	100	0	0	0	0
**Lokossa**	71	93.4	4	5.3	1	1.3	16	94.1	1	5.9	0	0

In addition to Hookworm, other STH groups including *Ascaris lumbricoides*, *Trichuris trichiura* and *Enterobius vermicularis* were identified. *A*. *lumbricoides* was found in 63% of the districts (19 out of 30 districts surveyed) with a prevalence ranging from 1% (95%CI: 0.0–2.2) to 39% (95%CI: 32.9–45.0) in endemic districts (Table 1). The severity of *A*. *lumbricoides* infestation is mostly light with some high intensity recorded in the districts of Lalo (23.5%), Toviklin (4.7%) and Djakotomey (4.7%) where the most infected child was found with 4,872 eggs counted i.e. over 100,000 eggs of *A*. *lumbricoides* per gram of faeces (Table 3). A total of 49 children were co-infected with both Hookworm and *A*. *lumbricoides*.

Cases of *Enterobius vermicularis*, *Trichuris trichiura* and *strongyloides stercoralis* were identified in many districts (Table 4). Of the 30 surveyed districts, *Enterobius vermicularis* was recorded in 21 districts with a prevalence ranging from 0.4% (95%CI: 0.0–1.2) to 15.2% (95%CI: 10.7–19.6). Only one pupil, an 11 years old girl in Kandi, was highly infected with an egg count of more than 5,000 for *E*. *vermicularis*. *Whipworm* was identified in 10 districts at low prevalence ranging between 0.8% (95%CI: 0.0–1.9) to 9.6% (95%CI: 5.9–13.2). All the other samples were lightly or moderately infected. There were no cases of high intensity infestations of whipworm. In Tchaourou and in Materi, moderate cases were identified while all the other positive samples were lightly infected. A total of 43 samples were found with light infection of *Strongyloides stercoralis*.

**Table 4:** Prevalence rate and intensity of other soil transmitted helminths found in the 30 districts mapped in 2014 in Benin. N: Total number of positive samples out the total (250) examined in each commune, 95%CI: 95% confidence interval for prevalence. For *E*. *vermicularis* and *T*. *trichiura intensity*: Light: 1-999EPG; Moderate: 1000-9999EPG; High: ≥10000EPG. For S. *stercoralis* Light: 1-800EPG; Moderate: 801-1200EPG; High: >1200EPG.

Districts	*Enterobius vermicularis*							*Trichuris trichiura*					*Strongyloides stercoralis*				
	Prevalence %[95%CI]	Light		Moderate		High		Prevalence % [95%CI]	Light		Moderate		Prevalence % [95%CI]	Light		High	
		N	%	N	%	N	%		N	%	N	%		N	%	N	%
**Banikoara**	0	0	0	0	0	0	0	1.2 [0.0–2.6]	3	100	0	0	0	0	0	0	0
**Gogounou**	6.8 [3.7–9.9]	17	100	0	0	0	0	0	0	0	0	0	0	0	0	0	0
**Malanville**	5.6 [2.7–8.5]	14	100	0	0	0	0	0	0	0	0	0	0	0	0	0	0
**Karimama**	9.6 [5.9–13.2]	24	100	0	0	0	0	0	0	0	0	0	0	0	0	0	0
**Segbana**	6.8 [3.7–9.9]	12	70.6	5	29.4	0	0	1.6 [1.0–3.2]	4	100	0	0	0	0	0	0	0
**Kandi**	19.2 [14.3–24.1]	41	87.2	6	12.8	1	2.1	0	0	0	0	0	0	0	0	0	0
**Kobli**	0	0	0	0	0	0	0	0	0	0	0	0	0	0	0	0	0
**Boukoumbe**	3.2 [1.0–5.4]	8	100	0	0	0	0	2.4 [0.5–4.3]	6	100	0	0	8.8 [5.3–12.3]	22	100	0	0
**Materi**	1.6 [1.0–3.2]	4	100	0	0	0	0	9.2 [5.6–12.8]	20	87.0	3	13.0	0	0	0	0	0
**Kouande**	0	0	0	0	0	0	0	0	0	0	0	0	0.4 [0.0–1.2]	1	100	0	0
**Toucountouna**	0	0	0	0	0	0	0	0	0	0	0	0	0	0	0	0	0
**Tanguieta**	12.0 [7.9–16.0]	25	83.3	5	16.7	0	0	0	0	0	0	0	0	0	0	0	0
**Tchaourou**	1.6 [1.0–3.2]	4	100	0	0	0	0	3.2 [1.0–5.4]	6	75.0	2	25.0	0	0	0	0	0
**Parakou**	2.8 [0.7–4.8]	7	100	0	0	0	0	0.8 [0.0–1.9]	2	100	0	0	0	0	0	0	0
**Sinende**	15.2 [10.7–19.6]	32	84.2	6	15.8	0	0	0	0	0	0	0	0	0	0	0	0
**Bante**	10.0 [6.3–13.7]	25	100	0	0	0	0	0	0	0	0	0	2.0 [0.3–3.7]	4	80	1	20
**Glazoue**	0	0	0	0	0	0	0	8.4 [4.9–11.8]	21	100	0	0	0	0	0	0	0
**Savalou**	0	0	0	0	0	0	0	2.0 [0.3–3.7]	5	100	0	0	1.6 [0.0–3.2]	4	100	0	0
**Aplahoue**	0.4 [0.0–1.2]	1	100	0	0	0	0	2.8 [0.7–4.8]	7	100	0	0	0	0	0	0	0
**Djakotomey**	5.6 [2.7–8.5]	14	100	0	0	0	0	0	0	0	0	0	0	0	0	0	0
**Dogbo**	0	0	0	0	0	0	0	9.2 [5.6–12.8]	22	91.7	1	4.2	0	0	0	0	0
**Klouekanmey**	3.6 [1.3–5.9]	9	100	0	0	0	0	0	0	0	0	0	0	0	0	0	0
**Lalo**	4.8 [2.1–7.5]	12	100	0	0	0	0	0	0	0	0	0	0	0	0	0	0
**Toviklin**	1.2 [0.0–2.6]	3	100	0	0	0	0	0	0	0	0	0	0	0	0	0	0
**Athieme**	0	0	0	0	0	0	0	0	0	0	0	0	0	0	0	0	0
**Bopa**	0	0	0	0	0	0	0	0	0	0	0	0	0	0	0	0	0
**Come**	4.0 [1.6–6.4]	10	100	0	0	0	0	4.8 [2.1–7.4]	12	100	0	0	4.4 [1.9–6.9]	11	100	0	0
**Grand Popo**	3.6 [1.3–5.9]	9	100	0	0	0	0	0	0	0	0	0	0	0	0	0	0
**Houeyogbe**	1.6 [0.0–3.2]	4	100	0	0	0	0	3.6 [1.3–5.9]	9	100	0	0	0	0	0	0	0
**Lokossa**	3.2 [1.0–5.4]	8	100	0	0	0	0	0	0	0	0	0	0	0	0	0	0

**Fig 2:**
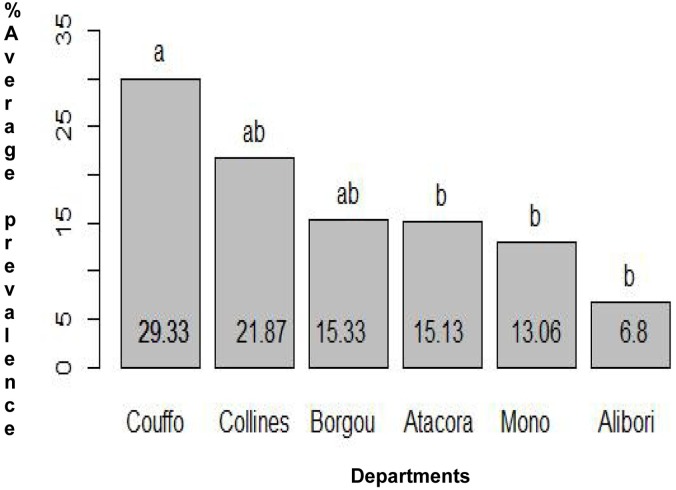
Comparison of the average prevalence of Hookworm in each department. On the chart, the department sharing similar letter are not significantly different. Those sharing different letters indicate a significant difference [p<0.0001]

**Fig 3:**
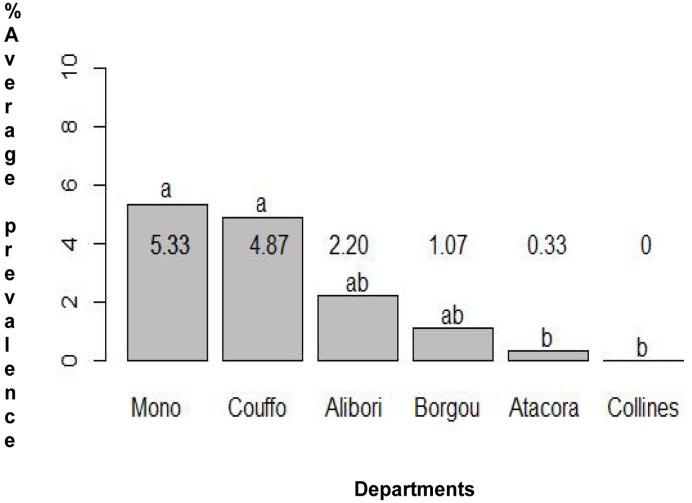
Comparison of the average prevalence of *A*. *lumbricoides* in each department.

A comparison of the Hookworm average prevalence per department indicated a significant difference between Couffo and all the other departments surveyed (Fig 2) and the departments of Mono and Couffo have a significant higher average prevalence for roundworm (Fig 3). A comparison within the district indicates that in Aplahoue, boys are twice more likely to be infected with Hookworm (OR: 2.2; 95%CI: 1.3–3.7) than girls in the districts of Parakou, Lokossa, Bante, Lalo and Come. In contrast, for the district of Kouande, boys were less likely to be infected with Hookworm compared to girls of similar age (OR: 0.37–95%CI: 0.18–0.74) (S2 Table). Due to the low number of positive samples of *E*. *vermicularis* and whipworm in most districts, data could not be subjected to powerful statistical analysis.

### Schistosomiasis distribution and intensity of infection

While *S*. *haematobium* was endemic in 29 districts, *S*. *mansoni* was identified in only 16 districts. In the department of Collines for example none of the districts surveyed was found with cases of *S*. *mansoni* although *S*. *haematobium* was endemic to all of them. There were no cases of schistosome infection detected in the district of Grand Popo during this survey.

The *S*. *haematobium* prevalence rate varied from 0.8% (95% CI: 0.0–1.9) to 56% (95% CI: 50.2–62.5) while the *S*. *mansoni* prevalence rate increased from 0.4% (95%CI: 0.0–1.2) to 46% (95% CI: 39.8–52.2) in districts where schistosome infection was detected. The district of Tanguieta had the lowest prevalence for *S*. *haematobium* but the highest prevalence rate for *S*. *mansoni*. On the contrary the district of Tchaourou had the highest prevalence rate for *S*. *haematobium* against 0.8% (95%CI: 0.0–1.9) for *S*. *mansoni*. Generally the prevalence of *S*. *haematobium* was either higher or similar to *S*. *mansoni* except in the districts of Lokossa, Materi and Tanguieta where the prevalence of *S*. *mansoni* was significantly higher than the prevalence of *S*. *haematobium* (Table 1).

**Fig 4:**
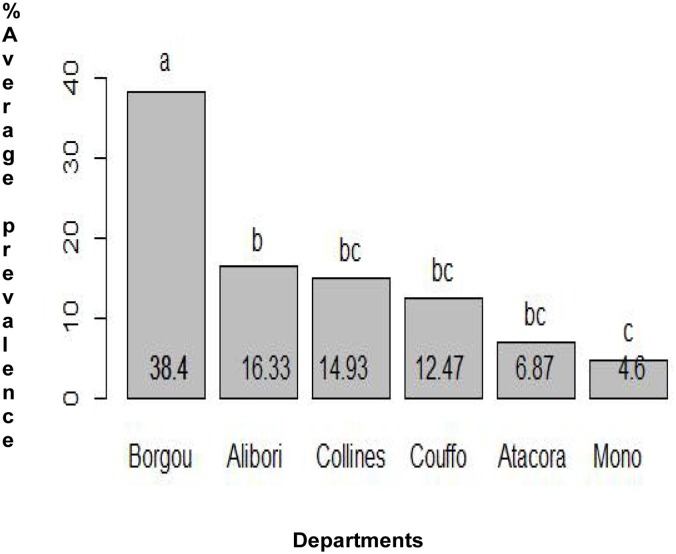
Comparison of the average prevalence of *S*. *haematobium* in each department.

**Fig 5:**
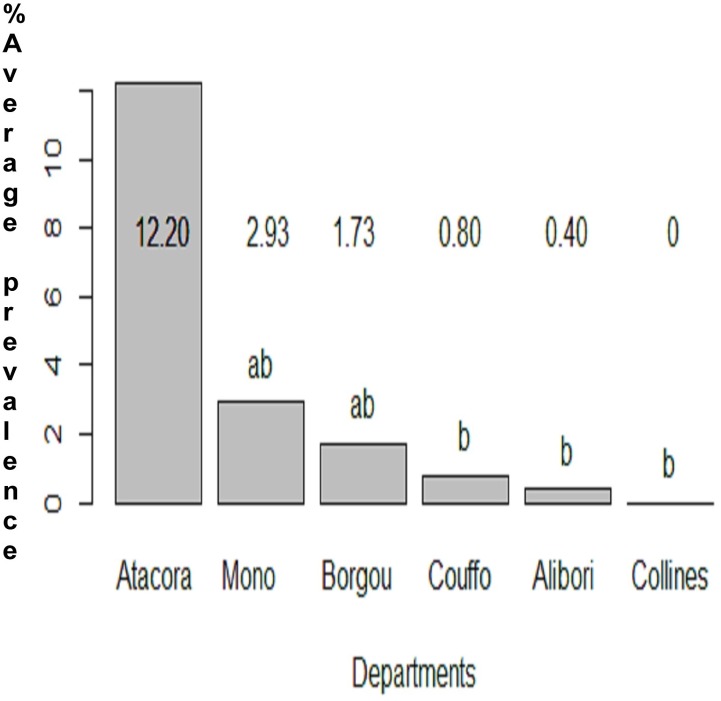
Comparison of the average prevalence of *S*. *mansoni* in each department.

Within the studied departments, the schistosomiasis was not uniformly distributed. The department of Borgou, where only two districts were surveyed, had the highest average prevalence of *S*. *haematobium* while Atacora was the department with the highest *S*. *mansoni* prevalence rate (Figs 4 & 5). In the majority of the surveyed districts, both urinary and intestinal schistosomiasis infection were not gender specific. However in the districts of Savalou, Klouekanmey and Boukoumbe, boys were likely to be infected with *S*. *haematobium* (S2 Table). A total 13 cases of *S haematobium* and *S*. *mansoni* co-infection were recorded throughout the study.

In most surveyed districts, the urinary schistosomiasis infestation was light (Table 5). Overall, 767 positive samples had 1–50 eggs of *S*. *haematobium* per 10 ml (light infestation) and 253 samples had more than 50 eggs of *S*. *haematobium* per 10 ml (high infestation). Similarly, the intestinal schistosomiasis infection was mostly light. A total of 207 positive samples had a light infection of 1–99 eggs of *S*. *mansoni* per gram of faeces, 102 positive samples with a moderate intensity of 100–399 eggs per gram and 63 samples with more than 400 eggs of *S*. *mansoni* per gram of faeces.

**Table 5:** Intensity of urinary and intestinal schistosomiasis infection in districts surveyed. N: Number of positive samples out the total (250) examined in each commune. For *S*. *haematobium* intensity Light: 1–50 eggs/10ml of urine; High: ≥ 50 eggs/10ml of urine For *S*. *mansoni* intensity Light: 1-99EPG; Moderate: 100–399 EPG; High: ≥ 400EPG.

District	*Schistosoma haematobium*				*Schistosoma mansoni*					
	Light		High		Light		Moderate		High	
	N	%	N	%	N	%	N	%	N	%
**Banikoara**	40	63.5	23	36.5	0	0	0	0	0	0
**Gogounou**	15	93.7	1	6.2	0	0	0	0	0	0
**Malanville**	80	100	0	0	1	100	0	0	0	0
**Karimama**	23	100	0	0	0	0	0	0	0	0
**Segbana**	2	40.0	3	60.0	2	40.0	3	60.0	0	0
**Kandi**	43	74.1	15	25.9	0	0	0	0	0	0
**Kobli**	11	78.6	3	21.4	94	93.1	6	5.9	1	1.0
**Boukoumbe**	28	56.0	22	44.0	19	41.3	18	39.1	9	19.6
**Materi**	2	28.6	5	71.4	8	50.0	7	43.7	1	6.2
**Kouande**	25	89.3	3	10.7	1	100	0	0	0	0
**Toukountouna**	1	50.0	1	50.0	0	0	0	0	0	0
**Tanguieta**	1	50.0	1	50.0	31	27.0	37	32.2	47	40.9
**Tchaourou**	82	58.2	65	41.8	2	10.0	14	70.0	4	20.0
**Parakou**	47	83.9	9	16.1	1	100	0	0	0	0
**Sinende**	82	90.1	9	9.9	9	90.0	1	10.00	0	0
**Bante**	30	65.2	16	34.8	0	0	0	0	0	0
**Glazoue**	15	93.7	1	6.2	0	0	0	0	0	0
**Savalou**	12	24.0	38	76.0	0	0	0	0	0	0
**Aplahoue**	47	100	0	0	1	100	0	0	0	0
**Djakotomey**	21	100	0	0	8	80.0	2	20.0	0	0
**Dogbo**	8	100	0	0	0	0	0	0	0	0
**Klouekanmey**	50	80.6	12	19.3	1	100	0	0	0	0
**Lalo**	35	72.9	13	27.1	0	0	0	0	0	0
**Toviklin**	11	100	0	0	0	0	0	0	0	0
**Athieme**	13	92.9	1	7.1	0	0	0	0	0	0
**Bopa**	1	50.0	1	50.0	4	100	0	0	0	0
**Come**	3	100	0	0	3	100	0	0	0	0
**Grand popo**	0	0	0	0	0	0	0	0	0	0
**Houeyogbe**	19	63.3	11	36.7	0	0		0		0
**Lokossa**	20	100	0	0	22	59.5	14	37.8	1	2.7

In addition to all these studied parasites, 95 samples with eggs of *Hymenolepis nana*, 24 with eggs of *Taenia spp* and seven samples with *Dicrocelium dendriticum* were identified at various levels of prevalence and intensity (S3 Table).

## Discussion

This study was a key study for the implementation of a well organised strategy to control schistosomiasis and STH in Benin. Prior to this mapping, sporadic drug administration for the control STH and schistosomiasis was carried out in some districts without a clear knowledge of the prevalence of the disease present there, in other words WHO guidelines on MDA [11] were not being followed. The mapping was scheduled in order to scale up MDA based on the prevalence of each endemic district. In addition, at a national level, there were no reports held on the treatment and the protocol used to map the country in 2003. Consequently there were no historical data available to assess the impact of treatment against schistosomiasis and STH in the endemic districts thus justifying the need for mapping. As a result of our study the mass drug administration for the control of schistosomiasis and STH has now being scaled up in accordance with the WHO guideline and the national policy. Benin national policy only accommodates one round of MDA a year even in districts that may require two rounds per year.

The current findings indicate STH endemicity in all surveyed districts at various levels depending on geographical location thus confirming that these parasite infections are endemic to all departments in Benin [27]. Apart from *A*. *lumbricoides* which seems to be predominant in the south eastern Benin, other STH infections were widely spread across the country with Hookworm being the most widely spread STH. *S*. *mansoni* and *S*. *haematobium* remain the only schistosome parasites identified in Benin so far [28,29]. The study has also confirmed that urinary schistosomiasis is more common than intestinal schistosomiasis in Benin [30,31]. Even though our study indicated that schistosomiasis and STH prevalence rates overall are higher than reported in a previous study [30,31], we believe Benin is following the trend of other Sub-Saharan African countries where the prevalence of these neglected tropical diseases (NTD) appears to drop over the time [32] and that schistosomiasis prevalence in Benin is steadily decreasing with time. As an example, in a previous study carried out among 1,585 pupils an average prevalence of 29% was observed [27] while the current average in the 30 districts for *S*. *haematobium* was less than 20%.

Among the districts surveyed, lymphatic filariasis was co-endemic in 22 out of the 30 and these 22 districts were included in the study in order to assess the impact of mass drug administration with an Albendazole and Ivermectin combination for LF. This had been carried out for many years in these districts and LF has even been cleared in some of these districts. We were expecting low STH prevalence in these districts as they have received six rounds of Albendazole treatment and LF has been eliminated successfully there. However there were no previous STH data available that could be used to compare with the current status. Nevertheless it is worth noting that in some of these districts such as Djakotomey (highest prevalence of STH) and Tchaourou (highest prevalence of schistosomiasis and only 19% for STH) LF-MDA was stopped just a year before this survey. On the one hand, this is an indication that school-based MDA would be needed even after LF elimination in districts where LF is endemic in order to reduce significantly STH prevalence in Benin. On the other hand however, this indicates that integrated control of diseases is essential. It is also a wake-up call for the Benin health system as it lacks strong prevention strategies and relies instead on MDA to control schistosomiasis. Currently, the national control program for these diseases is working in collaboration with donors who are only supporting MDA in Benin. However without any additional strategies, Benin will not be able to maintain treatment in endemic districts and this could lead to an increase of these infections.

The majority of the surveyed schools were fitted with some sort of modern lavatory accessible to the children, however, the first reflex of the children when they were asked to collect their faeces and urine, was to go to the wild (open air) indicating their usual habit. Children are not encouraged to use the lavatories even at school as the lavatories lack maintenance in almost all surveyed schools. We have noticed that many of these children walked to school bare foot even in urban areas, and are permanently exposed to the risk of contracting an STH infection. In such context, the national control program in charge of these diseases has set an epidemiological coverage target of 75% for school-based MDA. However, this target has not been reached in most districts as of 2014 and 2015, the epidemiological coverage rates were below 20%. In such an environment, where MDA coverage targets cannot be reached and where less than 50% of the population have access to adequate toilet facilities [33,34], coupled with the belief by some ethnic group of Benin that it is a sacrilege to use a toilet, the government needs to redesign an integrated approach for the control of schistosomiasis and STH and scale up sensitisation to raise awareness of these parasitic infections nationally. The National control program for communicable diseases is also concerned with the current situation and is planning a participative approach by evaluating the knowledge of the population in order to encourage them to initiate some preventive measures.

As this study was a baseline survey to determine whether a district requires schistosomiasis and STH MDA, only one stool and urine sample was collected from each selected student in the morning. This was done generally between 9 am and 10 am. However three specimens on three consecutive days could have been collected to assess daily egg shedding variations using the Formol ether concentration method for more accurate results. Due to logistical reasons this was not possible and the national control program was limited to one sample collection for each participant. In addition authors are aware of the fact that a single one-time Kato Katz specimen may not give confidence in the prevalence data given that the WHO recommends at least two averaged specimens. Further studies have therefore been scheduled in districts with no cases of schistosomiasis using the Formol ether concentration method on multiple samples.

## Conclusion

This disease mapping is a snapshot of what could be expected in other districts of Benin and has given the National Control Program of Communicable Disease of Benin baseline detail of the distribution of these neglected tropical diseases in endemic districts. The study will allow appropriate scale up of mass drug administration in endemic districts making the control of schistosomiasis and STH effective throughout the country. With adequate treatment and targets coverage, Benin should be able to control effectively these NTDs on its territory by 2020.

## Supporting Information

S1 Table Geographical position of the departments and all 150 schools survey during schistosomiasis and soil transmitted helminths mapping in 30 districts in Benin. UTM: Universal Transverse Mercator.
(PDF)

S2 Table Gender base comparison of schistosomiasis and Hookworm infection in the district surveyed. N: Total number sampled; OR: odds ratio; 95%CI [OR]: 95% Confidence interval for odds ratio; p(fisher): p-value of Fischer exact test for odds ratio; *S*.*h*: *Schistosoma haematobium; S*.*m*: *Schistosoma mansoni*
(PDF)

S3 Table Other parasites identified during schistosomiasis and soil transmitted helminths mapping in 30 districts in Benin. N: number of positive samples out the total (250) examined in each commune; 95%CI: 95% confidence interval for prevalence.
(PDF)

## References

[1] WHO. The prevention and control of schistosomiasis and soil transmitted helminthiasis. Report of a WHO Expert Committee. Geneva, WHO Technical Report Series No.912. 2002. 12592987

[2] De Silva NR, Brooker S, Hotez PJ, Montresor A, Engels D, Savioli L. Soil-transmitted helminth infections: updating the global picture. Trends Parasitol. 2003;19(12):547–551. 14642761 10.1016/j.pt.2003.10.002

[3] Hotez PJ, Bundy DA, Beegle K, Brooker S, Drake L, de Silva N, et al Helminth infections: soil-transmitted helminth infections and schistosomiasis. 2006 [cited 2015 Dec 21]; Available from: http://www.ncbi.nlm.nih.gov/books/NBK11748/

[4] Vos T, Flaxman AD, Naghavi M, Lozano R, Michaud C, Ezzati M, et al Years lived with disability (YLDs) for 1160 sequelae of 289 diseases and injuries 1990–2010: a systematic analysis for the Global Burden of Disease Study 2010. The Lancet. 2013;380(9859):2163–2196. 10.1016/S0140-6736(12)61729-2 PMC6350784 23245607

[5] WHO. Schistosomiasis, Fact Sheet No 115; World Health Organization. Available at http://www.who.int/mediacentre/factsheets/fs115/en/. 2015.

[6] Chitsulo L, Loverde P, Engels D. Schistosomiasis. Nat Rev Microbiol. 2004 1;2(1):12–3. 15035004 10.1038/nrmicro801

[7] Salawu AS, Asaolu SO, Sowemimo OA. Co-infections with *Schistosoma haematobium* and soil-transmitted helminths among school-aged children in Saki, Oyo State, Nigeria. J Public Health Epidemiol. 2014;6(12):417–423.

[8] Steinmann P, Keiser J, Bos R, Tanner M, Utzinger J. Schistosomiasis and water resources development: systematic review, meta-analysis, and estimates of people at risk. Lancet Infect Dis. 2006;6(7):411–425. 16790382 10.1016/S1473-3099(06)70521-7

[9] Van der Werf MJ, de Vlas SJ, Brooker S, Looman CW, Nagelkerke NJ, Habbema JD, Engels D. Quantification of clinical morbidity associated with schistosome infection in sub-Saharan Africa. Acta Trop. 2003 5;86(2–3):125–39. 12745133 10.1016/s0001-706x(03)00029-9

[10] Kheir MM, Eltoum IA, Saad AM, Ali MM, Baraka OZ, Homeida MM. Mortality due to schistosomiasis mansoni: a field study in Sudan. Am J Trop Med Hyg. 1999;60(2):307–10. 10072156 10.4269/ajtmh.1999.60.307

[11] WHO. Preventive chemotherapy in human helminthiasis: coordinated use of anthelminthic drugs in control interventions: a manual for health professionals and programme managers. World Health Organization Press, Geneva World Health Organization (2015), Schistosomiasis, A major public health problem http://www.who.int/schistosomiasis/en/. 2006.

[12] Brooker S, Clements AC, Bundy DA. Global epidemiology, ecology and control of soil-transmitted helminth infections. Adv Parasitol. 2006;62:221–261. 16647972 10.1016/S0065-308X(05)62007-6 PMC1976253

[13] Awasthi S, Bundy DA, Savioli L. Helminthic infections. Br Med J. 2003;327:431–433. 12933732 10.1136/bmj.327.7412.431 PMC188497

[14] Crompton DWT, Nesheim MC. Nutritional impact of intestinal helminthiasis during the human life cycle. Annu Rev Nutr. 2002;22(1):35–59. 12055337 10.1146/annurev.nutr.22.120501.134539

[15] Stephenson LS, Holland CV, Cooper ES. The public health significance of Trichuris trichiura. Parasitology. 2000;121(S1):S73–S95. 11386693 10.1017/s0031182000006867

[16] Nokes C, Cooper ES, Robinson BA, Bundy DA. Geohelminth infection and academic assessment in Jamaican children. Trans R Soc Trop Med Hyg. 1991;85(2):272–273. 1887491 10.1016/0035-9203(91)90052-z

[17] Nokes C, Bundy DAP. Does helminth infection affect mental processing and educational achievement? Parasitol Today. 1994;10(1):14–18. 15275558 10.1016/0169-4758(94)90348-4

[18] Jukes MC, Nokes CA, Alcock KJ, Lambo JK, Kihamia C, Ngorosho N, et al Heavy schistosomiasis associated with poor short-term memory and slower reaction times in Tanzanian schoolchildren. Trop Med Int Health. 2002;7(2):104–117. 11841700 10.1046/j.1365-3156.2002.00843.x

[19] Hotez PJ, Molyneux DH, Fenwick A, Kumaresan J, Sachs SE, Sachs JD, et al Control of Neglected Tropical Diseases. N Engl J Med. 2007;357(10):1018–27. 17804846 10.1056/NEJMra064142

[20] WHO. Schistosomiasis and soil-transmitted helminth infections. Geneva, World Health Organization, 2001. Resolution WHA54.19. 2001.

[21] Sady H, Al-Mekhlafi HM, Atroosh WM, Al-Delaimy AK, Nasr NA, Dawaki S, et al Knowledge, attitude, and practices towards schistosomiasis among rural population in Yemen. Parasit Vectors. 2015;8(1):1–13. 26302747 10.1186/s13071-015-1050-8 PMC4548916

[22] Alelign T, Degarege A, Erko B. Soil-Transmitted Helminth Infections and Associated Risk Factors among Schoolchildren in Durbete Town, Northwestern Ethiopia. J Parasitol Res [Internet]. 2015 [cited 2015 Dec 21];2015. Available from: http://www.hindawi.com/journals/jpr/2015/641602/abs/10.1155/2015/641602 PMC4487929 26161265

[23] WHO. Basic laboratory methods in medical Parasitology. Geneva, World Health Organization 1991.

[24] WHO_TRS_912.pdf [Internet]. [cited 2016 May 14]. Available from: http://apps.who.int/iris/bitstream/10665/42588/1/WHO_TRS_912.pdf

[25] Katz N, CHAVES A, Pellegring J. A simple device for quantitative stool thick-smear technique in *schistosomiasis mansoni*. 1972 [cited 2015 Dec 21]; Available from: http://en.journals.sid.ir/ViewPaper.aspx?ID=64869 4675644

[26] Tomas J. Aragon Developer (2012). epitools: Epidemiology Tools. R package version 0.5–7. https://CRAN.R-project.org/package=epitools

[27] Ibikounlé M, Gbédjissi LG, Ogouyèmi-Hounto A, Batcho W, Kindé-Gazard D, Massougbodji A. [Schistosomiasis and soil-transmitted helminthiasis among schoolchildren of Nikki and Perere, two northeastern towns of Benin]. Bull Soc Pathol Exot 1990. 2014;107(3):171–176. 10.1007/s13149-014-0344-y 24595888

[28] Moné H, Ibikounlé M, Massougbodji A, Mouahid G. Human schistosomiasis in the Economic Community of West African States: epidemiology and control. Adv Parasitol. 2010;71:33–91.

[29] Moné H, Minguez S, Ibikounlé M, Allienne J-F, Massougbodji A, Mouahid G. Natural Interactions between *S*. *haematobium* and *S*. *guineensis* in the Republic of Benin. Sci World J [Internet]. 2012 [cited 2015 Dec 21];2012. Available from: http://www.hindawi.com/journals/tswj/2012/793420/abs/10.1100/2012/793420 PMC3356739 22645454

[30] Chippaux JP, Massougbodji A, Zomadi A, Kindafodji BM. [Epidemiological study of schistosomes in a coastal lake of recent formation]. Bull Soc Pathol Exot 1990. 1989;83(4):498–508. 2126751

[31] Garba A, Kinde-Gazard D, Makoutode M, Boyer N, Ernould JC, Chippaux JP, et al [Preliminary evaluation of morbidity due to *S*. *haematobium* and *S*. *mansoni* in the area of the future Adjarala Dam in Benin]. Sante Montrouge Fr. 1999;10(5):323–328. 11125338

[32] Pullan RL, Smith JL, Jasrasaria R, Brooker SJ. Global numbers of infection and disease burden of soil transmitted helminth infections in 2010. Parasit Vectors. 2014;7(1):37. 24447578 10.1186/1756-3305-7-37 PMC3905661

[33] INSAE & ICF. Enquête Démographique et de Santé du Bénin 2011–2012 [Internet]. 2013 [cited 2015 Dec 21]. Available from: https://www.google.bj/search?newwindow=1&q=ICF+International%3A+Enque%CC%82te+De%CC%81mographique+et+de+Sante%CC%81+du+Be%CC%81nin+2011-2012&oq=ICF+International%3A+Enque%CC%82te+De%CC%81mographique+et+de+Sante%CC%81+du+Be%CC%81nin+2011-2012&gs_l=serp.3.2041.27869.0.28503.4.4.0.0.0.0.380.1323.2-1j3.4.0.0.1c.1.64.serp.1.3.940.wArQarihWT4

[34] Panesar A, Fink H, Glegbaza G, Kanathigoda A, Müller E. Sanitation activities in Benin. Bonn and Eschborn, Deutsche Gesellschaft für Internationale Zusammenarbeit (GIZ) Gmbh. 2015.

