# Supplementary material for: Schistosomiasis and Soil Transmitted Helminths Distribution in Benin: A Baseline Prevalence Survey in 30 Districts
Source: PLoS One. 2016 Sep 19;11(9):e0162798. doi: 10.1371/journal.pone.0162798 (PMC5028061; doi:10.1371/journal.pone.0162798)
Supplement: S1 Table — UTM: Universal Transverse Mercator. (PDF) [file pone.0162798.s001.pdf]

S1 Table: Geographical position of the departments and all 150 schools survey during schistosomiasis and soil transmitted helminths mapping in 30 districts in Benin. **UTM: Universal Transverse Mercator**

Department (UTM)	District	Sub-district	Public Primary School (PPS) surveyed	Altitude (meter)	Latitude	Longitude
**Alibori** (1319193.9N 530850.7E)	Banikoara	Kokey	PPS Kokey	308	11°22'50"N	2°31'21"E
		Banikoara	PPS Kommon	323	11°17'14"N	2°25'14"E
		Toura	PPS Atabenou	309	11°16'36"N	2°22'25"E
		Ounet	PPS Ounet	320	11°13'46"N	2°24'13"E
		Goumori	PPS Goumori	323	11°10'34"N	2°17'43"E
	Gogounou	Bagou	PPS Badou	321	10°53'55"N	2°44'6"E
		Sori	PPS Sori	343	10°43'1"N	2°46'56"E
		Borodarou	PPS Borodarou	300	10°58'44"N	2°52'29"E
		Wara	PPS Wara	360	10°40'34"N	2°40'25"E
		Wara	PPS Soukarou	363	10°41'23"N	2°41'36"E
	Malanville	Malanville	PPS Kotchi	191	11°51'22"N	3°25'20"E
		Tonmboutou	PPS Tonmboutou	204	11°51'27"N	3°17'20"E
		Malanville	PPS Houro Yesso	208	11°52'4"N	3°23'4"E
		Malanville	PPS Kambouwo Tounga	197	11°47'12"N	3°30'61"E
		Madekali	PPS Madekali	202	11°42'17"N	3°32'45"E
	Karimama	Monsey	PPS Monsey	190	12°17'29"N	2°55'20"E
		Kompa	PPS Kompa	178	12°12'13"N	3°1'59"E
		Tondikoaria	PPS Tondikoaria	195	12°1'57"N	3°12'25"E
		Karimama	PPS Hari Gani	198	12°3'59"N	3°10'53"E
		Birni Lafia	PPS Birni Lafia	196	11°58'50"N	3°13'11"E
	Segbana	Lougou	PPS Lougou	311	11°7'10"N	3°22'33"E
		Liboussou	PPS Liboussou	330	10°55'15"N	3°29'16"E
		Libante	PPS Libante	349	10°47'11"N	3°35'2"E
		Libante	PPS Saonzi	356	10°43'3"N	3°35'16"E
		Bobena	PPS Bobena	346	10°47'34"N	3°32'12"E
	Kandi	Angaradebou	PPS Angaradebou	312	11°19'0"N	3°2'9"E
		Angaradebou	PPS Thuy	307	11°17'21"N	3°1'25"E

		Kandi	PPS Kandi	316	11°8'20"N	2°56'11"E
		Kassakou	PPS Kassakou	320	11°4'56"N	2°54'8"E
		Kassakou	PPS Pade	296	11°2'28"N	2°53'0"E
**Atacora** (1188651.9N 354572.4E)	Cobly	Cobly	PPS Nanagade	229	10°28'32"N	0°54'24"E
		Cobly	PPS Nouagou	270	10°30'38"N	1°0'30"E
		Tapoga	PPS Tapoga	228	10°34'8"N	0°59'26"E
		Kountori	PPS Kountori	205	10°24'42"N	0°56'47"E
		Kountori	PPS Orou Kouare	240	10°22'48"N	0°59'37"E
	Boukoumbe	Manta	PPS Manta	242	10°20'57"N	1°6'34"E
		Boukoumbe	PPS Koutchagou	268	10°13'23"N	1°6'54"E
		Boukoumbe	PPS Koumontchirgou	257	10°7'35"N	1°7'58"E
		Boukoumbe	PPS Koutchata	239	10°11'26"N	1°6'12"E
		Boukoumbe	PPS Koutagou	272	10°8'50"N	1°8'21"E
	Materi	Nodi	PPS Nodi	237	10°37'51"N	1°1'37"E
		Dassari	PPS Dassari	219	10°48'38"N	1°8'37"E
		Tantega	PPS Koussega	234	10°49'38"N	1°0'14"E
		Gouande	PPS Gouande	187	10°46'44"N	0°54'58"E
		Tantega	PPS Porga	180	11°0'47"N	0°59'31"E
	Kouande	Sekougourou	PPS Sekougourou	426	10°22'52"N	1°45'21"E
		Fo Tance	PPS Fo Tance	434	10°24'58"N	1°41'31"E
		Birni	PPS Niarosson	484	10°11'59"N	1°36'56"E
		Guilmarou	PPS Guilmarou	461	10°33'50"N	1°43'41"E
		Kouboro	PPS Kouboro	383	10°3'16"N	1°33'13"E
	Toukountouna	Toukountouna	PPS Toukountouna	417	10°29'48"N	1°22'38"E
		Tchakalakou	PPS Tchakalakou	438	10°32'28"N	1°20'41"E
		Tchakifaga	PPS Tchakifaga	437	10°29'14"N	1°20'46"E
		Tampegre	PPS Nabaga	419	10°27'22"N	1°22'38"E
		Kouarfa	PPS Kouba	584	10°28'18"N	1°38'24"E
	Tanguieta	Tanguieta	PPS Tchoutchoubou	270	10°36'57"N	1°15'48"E
		Tayakou	PPS Makou	236	10°33'25"N	1°9'44"E
		Ndahonta	PPS Natagata	238	10°33'58"N	1°4'55"E

		Tanongou	PPS Tanongou	276	10°48'47"N	1°26'10"E
		Tanongou	PPS Batia	259	10°53'30"N	1°29'16"E
**Borgou** (1077781.9N 472580.4E)	Parakou	3rd Sub-district	PPS Wore	413	9°33'45"N	2°37'26"E
		1st Sub-district	PPS Dabou	374	9°27'3"N	2°33'59"E
		2nd Sub-district	PPS Korobororou	373	9°22'24"N	2°40'3"E
		1st Sub-district	PPS Bakperou	405	9°16'38"N	2°34'54"E
		1st Sub-district	PPS Tourou	397	9°20'53"N	2°32'54"E
	Sinende	Sinende	PPS Diadia	387	10°16'41"N	2°23'57"E
		Sinende	PPS Guessou Bani	374	10°20'3"N	2°16'4"E
		Sikki	PPS Siki	396	10°10'33"N	2°23'19"E
		Fo Boure	PPS Fo Sakarou	399	10°6'50"N	2°22'26"E
		Sekere	PPS Yarra	373	10°30'10"N	2°28'28"E
	Tchaourou	Beterou	PPS Beterou	294	9°12'2"N	2°16'23"E
		Alafiarou	PPS Agramarou	312	9°2'37"N	2°23'30"E
		Tchatchou	PPS Tchatchou	401	9°7'32"N	2°33'23"E
		Kika	PPS Kpassa	335	9°17'8"N	2°43'41"E
		Goro	PPS Goro Centre	351	8°58'32"N	2°29'39"E
**Collines** (884390.8N 408164.6E)	Glazoue	Ouedeme	PPS Ouedeme Centre	219	8°0'47"N	2°10'44"E
		Kpakpaza	PPS Sowe	201	7°58'47"N	2°9'58"E
		Aklamkpa	PPS Lagbo	227	8°13'23"N	2°11'5"E
		Zaffe	PPS Zaffe Centre	235	7°56'31"N	2°14'14"E
		Thio	PPS Riffo	181	8°4'44"N	2°19'0"E
	Savalou	Doume	PPS Doume	263	8°0'58"N	1°38'43"E
		Ouesse	PPS Akete	204	7°58'57"N	1°52'50"E
		Logozohe	PPS Logozohe	188	7°53'46"N	2°4'56"E
		Gobada	PPS Gobada	156	7°46'24"N	2°0'58"E
		Ottola	PPS Ottola	298	8°9'32"N	1°39'31"E
	Bante	Bobe	PPS Bobe	278	8°25'32"N	1°59'26"E
		Lougba	PPS Gotcha	274	8°15'57"N	1°44'16"E
		Pira	PPS Pira	348	8°29'40"N	1°43'48"E
		Bante	PPS Oladje	292	8°25'3"N	1°53'8"E

		Gouka	PPS Idjou	219	8°5'17"N	1°47'56"E
**Couffo** (727802.2N 387605.3E)	Klouekanmey	Pps Ehouzou A	PPS Couklo	196	6°58'45"N	1°50'11"E
		Ahogbeya	PPS Ahogbeya	179	7°1'13"N	1°54'4"E
		Adjahonme	PPS Sawame	204	7°5'53"N	1°46'49"E
		Lanta	PPS Tokamey Aliho	199	7°3'57"N	1°49'34"E
		Adjahoume	PPS Adjahoume	235	7°2'54"N	1°48'16"E
	Dogbo	Deve	PPS Deve	68	6°45'45"N	1°40'0"E
		Tota	PPS Dogbo Tota	98	6°48'17"N	1°47'2"E
		Ayomi	PPS Ayomi Centre	58	6°46'34"N	1°43'9"E
		Ayoni	PPS Kpodaha	120	6°49'31"N	1°42'34"E
		Madjre	PPS Madjre	104	6°49'32"N	1°50'47"E
	Aplahoue	Aplahoue	PPS Aplahoue Centre	172	6°56'26"N	1°40'38"E
		Kissamey	PPS Kissamey Centre	241	6°59'34"N	1°43'51"E
		Dekpo	PPS Dekpo	212	6°59'50"N	1°40'48"E
		Godohou	PPS Godohou	179	7°6'17"N	1°43'21"E
		Dekpo	PPS Dawui	215	7°1'35"N	1°40'40"E
	Djakotomey	Sokouhoue	PPS Sokouhoue	193	6°54'16"N	1°41'3"E
		Djakotomey	PPS Hagoumi	87	6°50'44"N	1°45'41"E
		Kinkinhoue	PPS Kinkinhoue	178	6°55'31"N	1°43'6"E
		Kpoba	PPS Kpoba Centre	116	6°50'25"N	1°39'22"E
		Kpoba	PPS Zohoudji Kpoba	111	6°49'17"N	1°38'19"E
	Lalo	Lokogba	PPS Lokogba	129	6°52'13"N	1°52'12"E
		Hlassame	PPS Kpassakanme	91	6°54'54"N	1°55'45"E
		Adoukandji	PPS Ahouada	61	6°53'26"N	1°59'5"E
		Ahomadagbe	PPS Ahomadegbe	47	6°52'11"N	2°0'36"E
		Ahomadagbe	PPS Alloya	69	6°51'29"N	1°58'35"E
	Toviklin	Toviklin	PPS Djigangnonhou	186	6°54'59"N	1°50'25"E
		Adjido	PPS Adjido	151	6°55'47"N	1°45'12"E
		Houedogli	PPS Houedogli	146	6°57'59"N	1°46'41"E
		Avedjin	PPS Dandjekoohoue	155	6°54'28"N	1°51'40"E
		Tannou Cola	PPS Tannou Cola	111	6°52'32"N	1°47'25"E

**Mono** (727838.4N 371020.4E)	Bopa	Lobogo	PPS Lobogo	124	6°37'23"N	1°54'30"E
		Agbodji	PPS Agbodji	32	6°38'27"N	1°58'52"E
		Gbakpodji	PPS Gbakpodji	45	6°39'56"N	1°51'9"E
		Yegodoe	PPS Yegodoe	59	6°43'29"N	1°52'58"E
		Badazouin	PPS Badazouin	63	6°34'18"N	1°56'6"E
	Athieme	Dedekpoe	PPS Dedekpoe	45	6°37'27"N	1°37'17"E
		Athieme	PPS Athieme	42	6°34'57"N	1°40'6"E
		Athieme	PPS Zounhoue	35	6°36'5"N	1°42'6"E
		Kpinnou	PPS Kpinnou	37	6°35'1"N	1°46'5"E
		Atchannou	PPS Atchannou	23	6°32'39"N	1°45'22"E
	Come	Agatogbo	PPS Agatogbo	30	6°24'12"N	1°55'20"E
		Guezin Kpota	PPS Guezin Kpota	41	6°23'17"N	1°57'1"E
		Akodeha	PPS Bowe Gbedji	64	6°27'47"N	1°54'59"E
		Ouedeme Pedah	PPS Ouedeme Pedah	58	6°29'27"N	1°56'38"E
		Agatogbo	PPS Kpetou	21	6°25'35"N	1°54'56"E
	Lokossa	Zoungame	PPS Zoungame	42	6°40'3"N	1°41'39"E
		Lokossa	PPS Urbaine Centre	62	6°37'55"N	1°42'44"E
		Ouedeme Adja	PPS Hlodo	44	6°43'16"N	1°40'4"E
		Houin	PPS Houin Dokodji	46	6°37'58"N	1°45'3"E
		Koudo	PPS Agnito	97	6°41'12"N	1°47'10"E
	Grand Popo	Grand Popo	PPS Ewe Condji	33	6°16'29"N	1°47'29"E
		Adjaha	PPS Adjaha	37	6°19'29"N	1°50'8"E
		Djanglanme	PPS Gountoeto	27	6°25'21"N	1°48'23"E
		Sague	PPS Sague	41	6°26'25"N	1°48'14"E
		Grand Popo	PPS Onkinhoue	13	6°16'35"N	1°48'17"E
	Houeyogbe	Houeyogbe	PPS Hounvi	80	6°32'35"N	1°51'12"E
		Zoungbonou	PPS Monhov	80	6°33'40"N	1°49'4"E
		Se	PPS Dre	57	6°27'39"N	1°50'9"E
		Doutou	PPS Tokpa	39	6°39'25"N	1°49'17"E
		Daye	PPS Daye	72	6°30'56"N	1°56'32"E
