# Supplementary material for: Schistosomiasis and Soil Transmitted Helminths Distribution in Benin: A Baseline Prevalence Survey in 30 Districts
Source: PLoS One. 2016 Sep 19;11(9):e0162798. doi: 10.1371/journal.pone.0162798 (PMC5028061; doi:10.1371/journal.pone.0162798)
Supplement: S2 Table — N: Total number sampled; OR: odds ratio; 95%CI [OR]: 95% Confidence interval for odds ratio; p(fisher): p-value of Fischer exact test for odds ratio; S.h: Schistosoma haematobium; S.m: Schistosoma mansoni (PDF) [file pone.0162798.s002.pdf]

S2 Table: Gender base comparison of schistosomiasis and Hookworm infection in the district surveyed. **N: Total number sampled; OR : odds ratio ; 95%CI [OR]: 95% Confidence interval for odds ratio ; p(fisher) : p-value of Fischer exact test for odds ratio; *S.h: Schistosoma haematobium; S.m: Schistosoma mansoni***

Districts	Sex	N Children	N cases of Hookworm	OR	95% IC[OR]	p(fisher)	N cases of *S. h.*	OR	95% IC[OR]	p(fisher)	N cases of *S.m.*	OR	95% IC[OR]	p(fisher)
Banikoara	M	124	10	1	-		37	1	-	-	-	-	-	-
	F	126	9	1.14	[0.39-3.29]	0.81	26	1.63	[0.88-3.05]	0.10	-	-	-	-
Gogounou	M	125	11	1	-		12	1	-	-	-	-	-	-
	F	125	15	0.71	[0.28-1.73]	0.53	4	3.19	[0.93-14.0]	0.06	-	-	-	-
Malanville	M	125	4	1	-		45	1	-	-	-	-	-	-
	F	125	3	1.34	[0.22-9.36]	1.0	35	1.44	[0.81-256]	0.22	1	-	-	-
Karimama	M	125	2	1	-		11	1	-	-	-	-	-	-
	F	125	1	2.01	[0.10-120]	1.0	12	0.91	[0.35-2.35]	1	-	-	-	-
Segbana	M	125	18	1	-		2	1	-	-	1	1	-	-
	F	125	17	1.07	[0.49-2.34]	1.00	3	0.66	[0.05-5.89]	1.00	4	0.24	[0.00-2.52]	0.37
Kandi	M	125	7	1	-		34	1	-	-	-	-	-	-
	F	125	5	1.42	[0.37-5.85]	0.76	24	1.56	[0.83-2.99]	0.18	-	-	-	-
Sinende	M	125	11	1	-		44	1	-	-	7	1	-	-
	F	125	3	3.90	[0.99-22.35]	0.05	47	0.90	[0.52-1.56]	0.79	3	2.40	[0.53-14.75]	0.33
Cobly	M	125	46	1	-		7	1	-	-	3	1		
	F	125	30	1.84	[1.03-3.32]	0.038	7	1.0	[0.29-3.45]	1.0	2	1.51	[0.17-18.37]	1.0
Boukoumbe	M	125	15	1	-		32	1	-	-	28	1		
	F	125	18	0.81	[0.36-1.80]	0.71	18	2.04	[1.03-4.13]	0.04	18	1.71	[0.85-3.51]	0.14
Materi	M	125	18	1	-		4	1	-	-	8	1	-	-
	F	125	9	2.16	[0.87-5.71]	0.10	3	1.34	[0.22-9.35]	1	8	1.0	[0.31-3.17]	1
Kouande	M	125	17	1	-		18	1	-	-	-	-	-	-
	F	125	37	0.37	[0.18-0.74]	0.003	10	1.93	[0.80-4.9]	0.16	1			
Toucountouna	M	125	8	1	-		2	1	-	-	-	-	-	-
	F	125	3	2.77	[0.64-16.6]	0.21		-	-	-	-	-	-	-

Tanguieta	M	125	15	1	-		2	1	-	-	56	1	-	-
	F	125	11	1.41	[0.58-3.56]	0.53		-	-	-	59	0.91	[0.53-1.54]	0.80
Tchaourou	M	124	29	1	-		73	1	-	-	-	-	-	-
	F	126	19	1.71	[0.86-3.46]	0.11	68	1.17	[0.69-1.99]	0.61	2	-	-	-
Parakou	M	125	37	1	-		36	1	-	-	-	-	-	-
	F	125	16	2.85	[1.44-5.88]	0.001	20	2.12	[1.10-4.15]	0.02	1	-	-	-
Bante	M	125	45	1	-		25	1	-	-	-	-	-	-
	F	125	16	3.81	[1.95-7.77]	0.000	21	1.24	[0.62-2.49]	0.63	-	-	-	-
Glazoue	M	125	34	1	-		10	1	-	-	-	-	-	-
	F	125	27	1.35	[0.73-2.53]	0.37	6	1.72	[0.55-5.95]	0.44	-	-	-	-
Savalou	M	125	24	1	-		29	1	-	-	-	-	-	-
	F	125	18	1.41	[0.69-2.94]	0.39	21	7.14	[3.25-16.19]	0.000	-	-	-	-
Aplahoue	M	125	51	1	-	-	26	1	-	-	1	-	-	-
	F	125	30	2.17	[1.22-3.91]	0.007	21	1.30	[0.65-2.60]	0.51	-	-	-	-
Djakotomey	M	125	77	1	-		12	1	-	-	3	1		
	F	125	73	1.14	[0.67-1.96]	0.69	9	1.37	[0.51-3.83]	0.65	7	0.42	[0.06-1.87]	0.33
Dogbo	M	120	2	1	-		3	1	-	-	-	-	-	-
	F	130	1	2.18	[0.11-129.9]	0.61	5	0.64	[0.09-3.38]	0.72	-	-	-	-
Klouekanmey	M	125	52	1	-		38	1	-	-	-	-	-	-
	F	125	44	1.31	[0.76-2.25]	0.36	24	1.83	[0.98-3.46]	0.06	1	-	-	-
Lalo	M	125	51	1	-		28	1	-	-	-	-	-	-
	F	125	22	3.21	[1.74-6.07]	0.000	20	1.51	[0.77-3.03]	0.26	-	-	-	-
Toviklin	M	125	21	1	-		1	1	-	-	-	-	-	-
	F	125	25	0.81	[0.40-1.61]	0.62		-	-	-	-	-	-	-
Athieme	M	125	15	1	-	-	8	1	-	-	-	-	-	-
	F	125	15	1	[0.43-2.31]	1	6	1.35	[0.39-4.89]	0.78	-	-	-	-
Bopa	M	125	12	1	-		1	1	-	-	2	1	-	
	F	125	10	1.22	[0.46-3.29]	0.82	1	1.0	[0.01-79.12]	1.0	2	1	[0.07-13.99]	1.00
Come	M	125	9	1	-		2	1	-	-	3	-	-	-
	F	125	1	9.55	[1.29-425.0]	0.019	1	2.0	[0.10-119.9]	1.0	-	-	-	-

Grand Popo	M	125	11	1	-			-	-	-	-	-	-	-
	F	125	9	1.24	[0.44-3.53]	0.81		-	-	-	-	-	-	-
Houeyogbe	M	125	24	1	-		14	1	-	-	-	-	-	-
	F	125	14	1.88	[0.88-4.16]	0.11	16	0.86	[0.37-1.98]	0.85	-	-	-	-
Lokossa	M	125	51	1			13	1	-	-	20	1	-	-
	F	125	25	2.74	[1.51-5.07]	0.000	7	1.95	[0.69-5.99]	024	17	1.21	[0.57-2.61	0.72
