# Supplementary material for: Schistosomiasis and Soil Transmitted Helminths Distribution in Benin: A Baseline Prevalence Survey in 30 Districts
Source: PLoS One. 2016 Sep 19;11(9):e0162798. doi: 10.1371/journal.pone.0162798 (PMC5028061; doi:10.1371/journal.pone.0162798)
Supplement: S3 Table — N: number of positive samples out the total (250) examined in each commune; 95%CI: 95% confidence interval for prevalence. (PDF) [file pone.0162798.s003.pdf]

S3 Table : Other parasites identified during schistosomiasis and soil transmitted helminths mapping in 30 districts in Benin. **N: number of positive samples out the total (250) examined in each commune; 95%CI: 95% confidence interval for prevalence**

Endemic districts	*Hymenolepis nana*		*Dicrocelium dentriticum*		*Taenia spp*	
	**N**	**Prevalence % [95%CI]**	**N**	**Prevalence % [95%CI]**	**N**	**Prevalence % [95%CI]**
Banikoara	6	2.4 [0.5-4.3]	2	0.8 [0.0-1.9]	0	0
Tanguieta	8	3.2 [1.0-5.4]	0	0	1	0.4 [0.0-1.2]
Toukountouna	6	2.4 [0.5-4.3]	4	1.6 [0.0-3.2]	11	4.4 [1.9-6.9]
Kouande	0	0	0	0	2	8.8 [5.3-12.3]
Materi	19	7.6 [4.3-10.9]	0	0	5	2 [0.3-3.7]
Cobly	7	2.8 [0.7-4.8]	0	0	2	0.4 [0.0-1.2]
Gogounou	4	1.6 [0.0-3.2]	0	0	0	0
Boukoumbe	8	3.2 [1.0-5.4]	0	0	0	0
Sinende	1	0.4 [0.0-1.2]	0	0	0	0
Parakou	0	0	0	0	1	0.4 [0.0-1.2]
Malanville	9	3.6 [1.3-5.9]	0	0	0	0
Karimama	0	0	0	0	2	0.8
Segbana	14	5.6 [2.7-8.4]	0	0	0	0
Tchaourou	2	0.8 [0.0-1.9]	0	0	0	0
Kandi	5	2 [0.3-3.7]	0	0	0	0
Savalou	1	0.4 [0.0 -1.2]	0	0	0	0
Lokossa	2	0.8 [0.0-1.9]	0	0	0	0
Bante	3	1.2 [0.0-2.5]	1	0.4 [0.0-1.2]	0	0
